# Transient Introgression of *Wolbachia* into *Aedes aegypti* Populations Does Not Elicit an Antibody Response to *Wolbachia* Surface Protein in Community Members

**DOI:** 10.3390/pathogens11050535

**Published:** 2022-05-03

**Authors:** Elvina Lee, Tran Hien Nguyen, Thu Yen Nguyen, Sinh Nam Vu, Nhu Duong Tran, Le Trung Nghia, Quang Mai Vien, Thanh Dong Nguyen, Robson Kriiger Loterio, Iñaki Iturbe-Ormaetxe, Heather A. Flores, Scott L. O’Neill, Duc Anh Dang, Cameron P. Simmons, Johanna E. Fraser

**Affiliations:** 1 Institute of Vector-Borne Disease, Monash University, Clayton, VIC 3800, Australia; elvina.m.h.lee@gmail.com (E.L.); robson.kriigerloterio@monash.edu (R.K.L.); heather.flores@monash.edu (H.A.F.); 2 National Institute of Hygiene and Epidemiology, Hanoi 100000, Vietnam; ngtrhien@yahoo.com (T.H.N.); yenanihe@yahoo.com (T.Y.N.); vusinhnam@gmail.com (S.N.V.); trannhuduong@gmail.com (N.D.T.); dangducanh.nihe@gmail.com (D.A.D.); 3 Institute Pasteur, Nha Trang 650000, Vietnam; nghiavbc@gmail.com (L.T.N.); vienquangmai@gmail.com (Q.M.V.); dongpasteur@gmail.com (T.D.N.); 4 World Mosquito Program, Institute of Vector-Borne Disease, Monash University, Clayton, VIC 3800, Australia; inaki.iturbe@worldmosquito.org (I.I.-O.); scott.oneill@worldmosquito.org (S.L.O.); cameron.simmons@worldmosquito.org (C.P.S.); 5 Oxford University Clinical Research Unit, Ho Chi Minh City 710400, Vietnam; 6 Nuffield Department of Medicine, University of Oxford, Oxford OX3 7BN, UK; 7 Department of Microbiology, Monash Biomedicine Discovery Institute, Monash University, Clayton, VIC 3800, Australia

**Keywords:** *Wolbachia*, *Aedes aegypti*, vector biology, arbovirus, dengue virus

## Abstract

*Wolbachia* is an endosymbiotic bacterium that can restrict the transmission of human pathogenic viruses by *Aedes aegypti* mosquitoes. Recent field trials have shown that dengue incidence is significantly reduced when *Wolbachia* is introgressed into the local *Ae. aegypti* population. Female *Ae. aegypti* are anautogenous and feed on human blood to produce viable eggs. Herein, we tested whether people who reside on Tri Nguyen Island (TNI), Vietnam developed antibodies to *Wolbachia* Surface Protein (WSP) following release of *Wolbachia*-infected *Ae. aegypti*, as a measure of exposure to *Wolbachia*. Paired blood samples were collected from 105 participants before and after mosquito releases and anti-WSP titres were measured by ELISA. We determined no change in anti-WSP titres after ~30 weeks of high levels of *Wolbachia*-*Ae. aegypti* on TNI. These data suggest that humans are not exposed to the major *Wolbachia* surface antigen, WSP, following introgression of *Wolbachia*-infected *Ae. aegypti* mosquitoes.

## 1. Introduction

*Aedes aegypti* mosquitoes are the major vector for many human pathogenic viruses, including dengue, Zika, and chikungunya. Dengue virus prevalence has risen sharply in recent decades, driven in part by the adaptation of these mosquitoes to thrive in densely populated mega-cities [1,2]. Despite the global burden of dengue, there is no approved antiviral therapeutic to treat infections, and uptake of the one approved vaccine has been poor [3]. As such, vector control methods remain the major defence employed to limit the spread of dengue virus.

*Wolbachia pipientis* is an intracellular, endosymbiotic bacterium that has been developed as a biocontrol tool to reduce the vector competence of *Ae. aegypti*. Several *Wolbachia* strains have been transinfected into *Ae. aegypti* and shown to reduce the risk of transmission of flavi- and alphaviruses [4,5,6,7,8,9]. Introgression of the *Wolbachia* strain *w*Mel (from *Drosophila melanogaster*) into *Ae. aegypti* populations significantly reduces the incidence of dengue and chikungunya in humans [10,11,12,13,14].

*Ae. aegypti* are anautogenous, strictly requiring a blood meal in order to lay viable eggs, and humans are predominantly their meal source [15]. This direct interaction, whereby mosquitoes expel saliva as they probe for a blood vessel, could potentially provide an opportunity for humans to be exposed to *Wolbachia* antigens. *Wolbachia* surface protein (WSP) is a major surface membrane protein exposed to its host and therefore may represent the main antigenic target. Previous studies have attempted to measure *Wolbachia* in the saliva of *Ae. aegypti* with a *Wolbachia* infection [16] and antibodies to *Wolbachia* in the plasma of people who routinely feed lab colonies of *Wolbachia*-*Ae. aegypti* [17]. However, no study to date has directly investigated the potential for *Wolbachia* exposure to residents at field sites where these mosquitoes are introgressed.

In 2013, the *Wolbachia* strain *w*MelPop-CLA (originally from *D. melanogaster*, then adapted to the mosquito host by long-term passage through two *Ae. albopictus* cell lines [18]; referred to herein as *w*MelPop) was assessed for its ability to introgress into *Ae. aegypti* populations at sites in Northern Australia and central Vietnam [19]. Herein, we examine whether this introgression of *Wolbachia*-*Ae. aegypti* into local mosquito populations caused residents in Vietnam to develop antibodies to WSP as a measure of *Wolbachia* exposure. Paired blood samples were collected pre- and post-release of *w*MelPop-*Ae. aegypti* from 105 residents of Tri Nguyen Island (TNI) in Khanh Hoa Province, Vietnam. We measured the IgA/G/M antibodies to *Wolbachia* WSP in plasma from these participants using a new enzyme-linked immunosorbent assay (ELISA). Our data support the hypothesis that humans are not exposed to *Wolbachia* antigens when bitten by *Ae. aegypti* infected with *w*MelPop.

## 2. Results

### 2.1. Enrolment of Participants on Tri Nguyen Island

**Figure 1:**
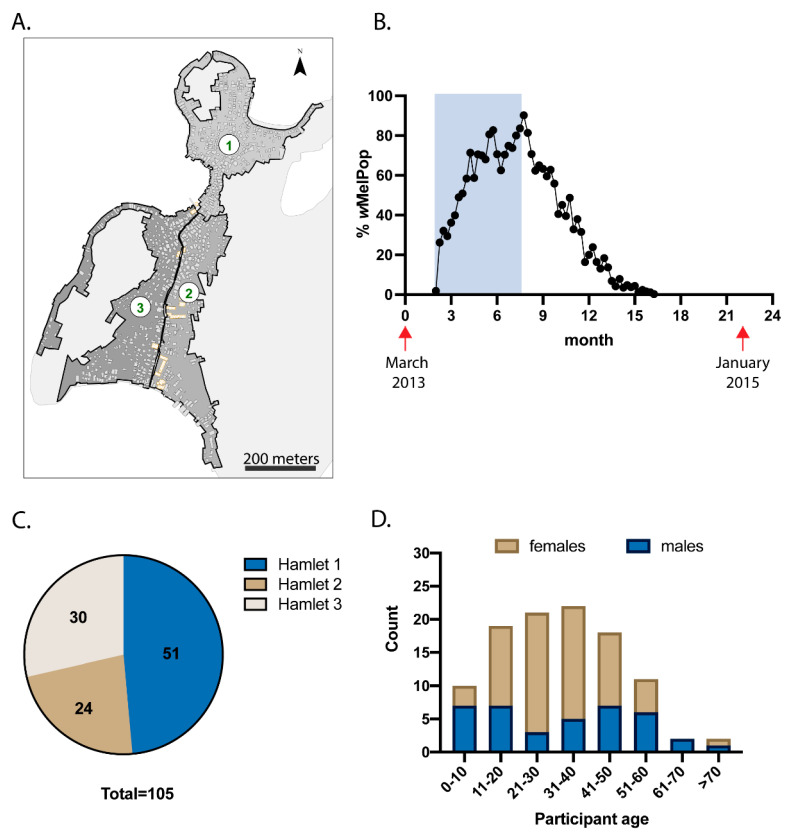
Participant demographics. (**A**) Releases of *Ae. aegypti* with *w*MelPop strain of *Wolbachia* were performed across 3 hamlets on TNI, Vietnam. (**B**) *w*MelPop-*Ae. aegypti* were released weekly for 23 weeks (shaded rectangle), and routine trapping of mosquitoes was performed using Biogents Sentinel traps to monitor the proportion of *Ae. aegypti* with *w*MelPop infection (plotted in black). Plasma was collected from 105 participants prior to mosquito releases, then collected again 22 months later (collection times indicated by red arrows). Participant breakdown by hamlet location (**C**) and by age and gender (**D**) is shown.

As reported previously [19], in 2013, *w*MelPop-*Ae. aegypti* were released for 23 consecutive weeks across three hamlets on TNI, Khanh Hoa Province, Vietnam [19] (Figure 1A,B shaded area). At its peak, this *Wolbachia* strain was found in approximately 90% of the *Ae. aegypti* population (Figure 1B). However, when releases ceased, *w*MelPop frequency declined rapidly, most likely due to fitness costs incurred by the mosquito [19]. Despite this, residents of TNI experienced nearly 7 months when *w*MelPop-*Ae. aegypti* frequency was > 50% of the total *Ae. aegypti* population. To determine whether residents are exposed to *Wolbachia* when these biting mosquitoes are established in the local environment, baseline plasma samples were collected one month prior to commencement of mosquito releases, and then again 22 months later (Figure 1B, red arrows). Plasma samples from 105 participants were assessed in this study, including 51 from hamlet 1, 24 from hamlet 2, and 30 from hamlet 3 (Figure 1C). Participants’ ages ranged from 1 to 76 years at the time of enrolment, and a total of 38 male participants and 67 female participants were studied (Figure 1D).

### 2.2. Establishment of an Enzyme-Linked Immunosorbent Assay (ELISA) to Detect Plasma Antibodies with Reactivity to WSP

We developed an ELISA to measure plasma IgA/G/M against WSP to assess whether individuals are exposed to *Wolbachia* following establishment of these mosquitoes in the local environment. Recombinant WSP residues 33-201 (WSP_33-201_) representing soluble *w*MelPop WSP (identical in sequence to *w*Mel WSP, Figure S1A and [20]) were expressed in *E. coli* with a 10× His tag to facilitate purification. The purified protein (Figure S1B) was used to raise polyclonal antibodies to WSP in rabbits. Anti-WSP polyclonal antibodies were shown to bind to both *w*Mel and *w*MelPop WSP from *Ae. aegypti*-derived Aag2 cells (Figure S1C,D).

**Figure 2:**
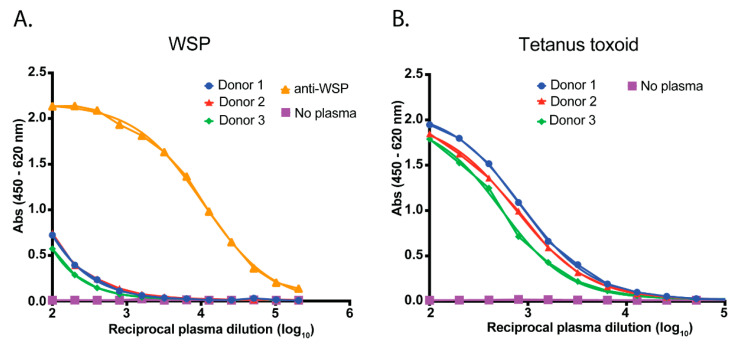
Assay validation. (**A**) A direct ELISA was established to measure human plasma IgA/G/M reactivity to WSP. Plasma from donors located in southern regions of Australia (where *Ae. aegypti* with *Wolbachia* are not found) were used as negative controls. These plasmas were pooled and then spiked with polyclonal anti-WSP to generate a positive serological signal in the assay. (**B**) In a parallel ELISA, plasma samples were tested for antibody reactivity to tetanus toxoid protein to demonstrate the integrity of the plasma samples for serological purposes. Data have been fitted with a sigmoidal dose-response (variable slope) curve using GraphPad Prism^®^ software (PRISM 7 version, GraphPad Software Inc., San Diego, CA, USA).

A direct ELISA was established using WSP_33-201_ to coat 96-well immunosorbent plates. Plasma from blood donors located in southern regions of Australia, where *Wolbachia*-*Ae. aegypti* are not found, was used to determine the background reactivity of human plasma to WSP. Reactivity was shown to be very low compared to plasma spiked with anti-WSP (Figure 2A). To validate the integrity of the plasma samples, we also ran a parallel ELISA using tetanus toxoid protein to confirm the presence of anti-tetanus toxoid IgA/G/M (Figure 2B). This antigen was selected due to the high global vaccination rate against tetanus, including in Vietnam and Australia [21,22]. Each of the three donor plasmas demonstrated strong reactivity to this control antigen.

### 2.3. Release of wMelPop-Ae. aegypti Does Not Alter Plasma Immunoreactivity to WSP

To determine whether individuals developed antibodies to *Wolbachia* after exposure to *w*MelPop-*Ae. aegypti*, paired plasma samples from 105 TNI residents were titrated across immunosorbent plates that had been coated with WSP_33-201_. Plasma samples from 29 Red Cross blood donors located in southern regions of Australia were included as individual negative controls for *Wolbachia* serology. In parallel, each plasma sample was also tested for reactivity to tetanus toxoid.

**Figure 3:**
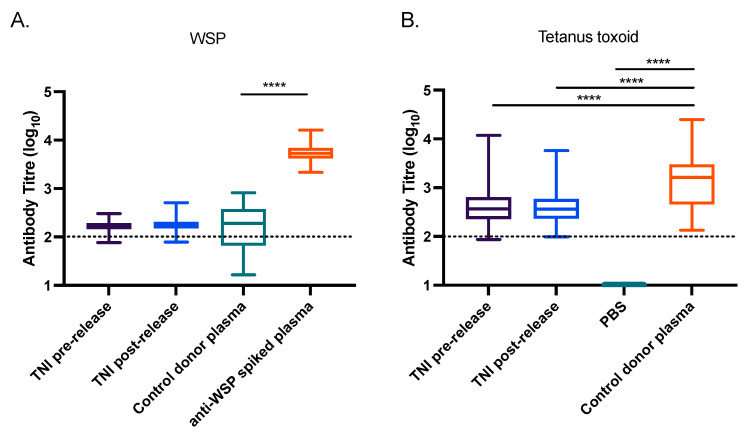
Introgression of *Wolbachia* into *Ae. aegypti* mosquito populations does not induce a detectable anti-*Wolbachia* immune response in humans. (**A**,**B**) Plasma from 105 donors living on TNI, Vietnam, pre- and post-release of *w*MelPop-*Ae. aegypti* were screened for antibodies to WSP (**A**), or tetanus toxoid (**B**) by ELISA. Australian plasma collected from 29 Red Cross donors in regions where *Wolbachia*-*Ae. aegypti* are not found were also assessed (control donor plasma). This served as a negative control for the WSP ELISA, and a positive control for the tetanus toxoid ELISA. A pool of 5 negative control donor plasmas spiked with polyclonal anti-WSP was used to indicate a positive serological signal for the WSP ELISA, while PBS was included as a negative control for the tetanus toxoid ELISA. The antibody titre for each sample is expressed as the reciprocal of the highest dilution of plasma required to achieve an absorbance value of 0.4, determined by nonlinear regression analysis performed using GraphPad Prism^®^ software (PRISM 7 version, GraphPad Software Inc., San Diego, CA, USA). Data are the median, interquartile range, minimum, and maximum antibody titres for each cohort. Asterisks indicate significance, determined by a Kruskal-Wallis test with Dunn’s correction, where **** indicates *p* < 0.0001. The minimum measurable antibody titre for each antigen is indicated by a dotted line.

Using a paired *t*-test, we measured no significant difference in the anti-WSP titres in plasma from individuals before or after exposure to *w*MelPop-*Ae. aegypti* (Figure 3A). The median anti-WSP titres for these groups were also not significantly different from the negative control plasmas (Kruskal-Wallis test with Dunn’s correction). As expected, the median anti-WSP-spiked plasma titre was significantly higher than the negative control plasmas (*p* < 0.0001), demonstrating that the assay had the capacity to measure antibodies to WSP. Plasma from the TNI participants also did not significantly change in reactivity to tetanus toxoid between the pre- and post-release plasma samples (paired *t*-test; Figure 3B). The Australian Red Cross donor control plasmas had significantly higher reactivity to tetanus toxoid compared to a PBS negative control (Kruskal-Wallis test with Dunn’s correction, *p* < 0.0001). The median titre of anti-tetanus toxoid was also slightly but significantly higher in the Red Cross control plasmas compared to the TNI pre- and post-release groups (*p* < 0.0001).

Breakdown of the data from Figure 3A to directly compare the TNI pre- and post-release plasma samples for each individual participant identified no clear trend in increasing or decreasing anti-WSP IgG/A/M titres following release of *w*MelPop-*Ae. aegypti* (Figure S2). Together, these data suggest that environmental exposure to *w*MelPop-*Ae. aegypti* does not induce a detectable level of antibodies to the *Wolbachia* antigen WSP in local residents.

## 3. Discussion

*Wolbachia* naturally resides as a non-pathogenic endosymbiont in a wide range of invertebrates [23,24,25], including in human-biting mosquito species *Ae. albopictus* (*w*AlbA and *w*AlbB) [26,27] and *Culex quinquefasciatus* (*w*Pip) [23]. *Wolbachia* strains found in insect species are not considered pathogens of vertebrate animals. However, distantly related *Wolbachia* species from nematodes may contribute to filariasis pathogenesis [28,29,30,31,32]. With the recent development of population replacement methods introgressing *Ae. aegypti* with *Wolbachia* into mosquito populations, here we ask whether residents become seropositive to the antigenic *Wolbachia* protein, WSP, following their introgression.

Our findings build on the safety profile for the use of *Wolbachia* as a biocontrol tool in *Ae. aegypti* [16,17,33,34]. The results indicate that residents of TNI did not develop a measurable antibody response to WSP following the transient establishment of *w*MelPop-*Ae. aegypti* in their local area. This is consistent with evidence that *Wolbachia* is not detectably transferred through mosquito saliva. For example, Moreira et al. (2009) collected saliva expectorated from *w*MelPop-*Ae. aegypti* and attempted to amplify *Wolbachia* genes *WSP* and the multi-copy transposable element *IS5*, but were unable to detect either *Wolbachia* gene [16]. Popovici et al. (2010) investigated whether human volunteers that routinely allowed *Wolbachia*-*Ae. aegypti* colonies to directly feed on their arms developed antibodies against *w*MelPop [17]. To do this, they analysed sera from 17 volunteers who had fed mosquito colonies over a 4-year period, using Western blots and ELISAs with *Wolbachia*-infected or -uninfected cell extracts used as antigen. IgG reactivity to *Wolbachia* was not found to be different compared to volunteers who had not previously fed *Wolbachia*-infected *Ae. aegypti* mosquitoes, whilst blood-feeder volunteers (who may be exposed to tens of thousands of mosquito bites over 4 years) developed antibodies to a 45 kDa *Ae. aegypti* protein. While our findings are consistent with these previous studies, we acknowledge that the collection of plasma from residents occurred ~5 months after *w*MelPop was no longer detectable in the local *Ae. aegypti* population. We therefore cannot exclude the possibility that a short-lived antibody response to *Wolbachia* was induced.

Mosquito salivary proteins are well-known to induce an IgG response in humans [35,36,37], which occurs following salivation of mosquitoes as they probe the dermal layer for blood vessels. This probing and salivation can lead to the transfer of pathogenic viruses from infected mosquitoes into humans or other animals [38]. However, since *Wolbachia* is an obligate intracellular bacterium, this would require it to be released from host mosquito cells, secreted into the lumen of the salivary gland duct, and transferred through the proboscis of the mosquito. If *Wolbachia* were released from host cells, transfer may be further restricted by the diameter of the salivary gland ducts (1–2 μm [39]), which may not readily allow passage of *Wolbachia* (up to 1.5 μm in diameter for *w*Mel, *w*AlbB, and *w*MelPop [40,41,42,43]).

Most of the studies investigating the risk of *Wolbachia* transfer from mosquitoes to humans have assessed *w*MelPop, while current field trials are primarily using *w*Mel or *w*AlbB *Wolbachia* strains. While *w*MelPop has been introgressed into *Ae. aegypti* populations at field sites in both Vietnam and Northern Queensland (Australia), it has not remained stable, eventually dropping out [19]. By contrast, *w*Mel and *w*AlbB have remained at high frequency following introgression into mosquito populations at multiple sites, potentially exposing residents to these strains of *Wolbachia* for much longer periods of time [11,13,44,45]. It is therefore important to consider whether these strains similarly present the same low risk. All three strains are distributed throughout a range of mosquito tissues, including the ovaries, midgut, malpighian tubules, and salivary glands [9,46]. However, *w*MelPop resides at a higher overall density in whole mosquitoes than *w*Mel and *w*AlbB [47], which might suggest that the risk of transfer for these strains is lower.

Whilst we were unable to detect human Ig responses to WSP, it is possible that other *Wolbachia* antigens do enter the mosquito saliva, perhaps following their secretion into host cells via the bacterial type IV secretion system [48,49]. We therefore cannot exclude the possibility that these products induce an immune response in humans. In regions endemic for the nematode *Brugia malayi* (which parasitises human hosts), residents develop anti-WSP antibodies as well as antibodies that recognise the *Wolbachia* secreted protein *Wolbachia* Translation Initiation Factor-1 [50]. During *Onchocerca volvulus* (another human-parasitic nematode) infection, *Wolbachia* lipopeptide can induce the production of Neutrophil Extracellular Traps (an anti-microbial defence mechanism) [51]. Additionally, release of *Wolbachia* into patients, following Ivermectin treatment to clear *O. volvulus* infections, correlates with post-treatment inflammation events [52]. These studies indicate that various *Wolbachia* products may be able to induce an immune response. However, dozens of adult nematodes and thousands of microfilariae are present in human infections [53,54], with each nematode cell carrying multiple *Wolbachia* [55]. Therefore, the human exposure to *Wolbachia* components from nematodes may be magnitudes higher than the exposure that may hypothetically occur from *Wolbachia* components present in the saliva of *Wolbachia*-infected *Ae. aegypti*.

Interestingly, Punkodsy et al. (2003) reported that 15 of 67 serum samples from individuals from North America with no history of lymphatic filariasis were seropositive to WSP [32]. These antibodies recognized a highly conserved region of the second transmembrane domain of WSP, perhaps suggesting that these people were exposed to *Wolbachia* from other non-nematode species. Notably, *Wolbachia* strains from nematodes cluster phylogenetically to supergroups C and D, and are mostly obligate mutualists [56]; curing nematodes of their *Wolbachia* infection by antibiotic treatment leads to nematode death [57]. By contrast, supergroup A and B *Wolbachia* strains, including *w*Mel, *w*MelPop, and *w*AlbB, are usually found in arthropods, and their relationship is not required for the survival of the host [58]. Additionally, *Wolbachia* species from nematodes survive at their host temperature of 37 °C compared to the cooler temperatures habited by *Wolbachia* from arthropods. This clear distinction in phylogeny and host relationship for *Wolbachia* strains found in nematodes compared to arthropods could predict that arthropod-derived *Wolbachia* strains may be unable to establish infection in humans, even if exposure events do occur.

It should be acknowledged that a 2015 study detected *Wolbachia* DNA in the blood of a patient subsequently diagnosed with non-Hodgkin’s lymphoma, with the authors proposing a possible novel pathogenic infection by *Wolbachia* alone [59]. *Wolbachia 16S* and *fbpA* genes were amplified from the infected patient’s blood and found to cluster most closely with *Wolbachia* supergroup B strains. This is consistent with *Wolbachia* species from insects. No causative agent was isolated, and there have been no further reports of possible direct human infections with *Wolbachia*, so the significance of this study remains unclear.

With several recent publications demonstrating the efficacy of *Wolbachia*-*Ae. aegypti* introgression programs in reducing vector-borne disease, it is likely that a global expansion of these programs will occur in coming years [10,11,12,13,14]. Our evidence indicates that transient introgression of *w*MelPop into *Ae. aegypti* populations did not seroconvert residents to the major *Wolbachia* antigen, WSP. These data add to the body of evidence that *Wolbachia* is a safe biocontrol tool to limit the spread of mosquito-borne viruses.

## 4. Materials and Methods

### 4.1. Recruitment of Blood Donors on Tri Nguyen Island, Vietnam

The release of *Ae. aegypti* with *w*MelPop at Tri Nguyen Island (TNI) and recruitment of volunteers for plasma donations was approved by the institutional review board (IRB) of the National Institute of Hygiene and Epidemiology (Approval reference number: 32/HDD 15/12/2011) and the IRB of Vietnam Ministry of Health (Approval reference number: 615/CN-BYT 19/7/2012).

Blood samples were collected from residents in each of the 3 hamlets of TNI as part of general health surveys before and after the release of *Ae. aegypti* with *w*MelPop. These samples were used to measure changes in plasma reactivity to *Wolbachia* before and after intervention (i.e., release of *Wolbachia*-*Ae. aegypti*), similar to an interrupted time series study design. Informed consent to participate was obtained from all participants aged over 18 and from parents/guardians for those under 18. Baseline plasma samples were collected from residents in March 2013, before release of any *w*MelPop-*Ae. aegypti*. Beginning in April 2013, mosquitoes were released weekly for 23 weeks, as described previously [19]. The proportion of *Ae. aegypti* infected with *w*MelPop increased throughout the release period, peaking at ~90% of the total *Ae. aegypti* population by week 23. However, immediately after releases stopped, the frequency of mosquitoes with *w*MelPop declined rapidly, with fewer than 10% of *Ae. aegypti* carrying *w*MelPop by week 47 [19]. Overall, it is expected that residents had ~30 weeks of exposure to *w*MelPop-*Ae. aegypti* where these mosquitoes comprised > 50% of the *Ae. aegypti* population. In January 2015, repeat plasma collections were taken from the same individuals. Overall, 142 paired plasma samples were collected from residents (pre- and post-release of *w*MelPop-*Ae. aegypti*), and 105 of these were randomly chosen to be assayed here. Participant ages ranged from 1 to 76 years at the time of the first collection.

Plasma was extracted by centrifuging whole blood at 290× *g* for 10 min. The upper plasma layer was collected and heat inactivated at 56 °C for 30 min.

### 4.2. Control Plasmas

Red Cross blood from donors in Australian states where *Ae. aegypti* mosquitoes are not found (Victoria and South Australia) were used as negative controls for the ELISA assay that measured *Wolbachia* immunogenicity. Plasma from 29 donors was extracted from whole blood and heat inactivated, as described above.

### 4.3. WSP Overexpression and Purification

The WSP sequence from *w*Mel and *w*MelPop are identical (Figure S1A). *w*MelPop-CLA is a variant of *w*MelPop that was generated by passage of *w*MelPop through mosquito cell lines for ~3.5 years [18]. This variant acquired a suite of genomic changes outside of the WSP gene (*WD_1063*) and was then transinfected into *Ae. aegypti*, where the genome remained quite stable [20]. The sequence of WSP from *w*MelPop-CLA is identical to both *w*Mel and *w*MelPop. Note that *w*MelPop-CLA from transinfected *Ae. aegypti* has previously been referred to as *w*MelPop-PGYP in other publications [19,20]. For simplicity, we refer to *w*MelPop-CLA throughout this manuscript as *w*MelPop.

WSP from *w*Mel was expressed and purified by The Commonwealth Scientific and Industrial Research Organisation (CSIRO; Parkville, Melbourne, Australia) in a truncated form; the N-terminal 33 amino acids and C-terminal 36 amino acids were removed to promote stability and solubilization. WSP residues 33–201 were expressed with an N-terminal 10× His tag to facilitate purification.

Briefly, the truncated WSP nucleotide sequence was cloned into the pD434-SR *E. coli* expression vector (ATUM, Newark, CA, USA). BL21(DE3) cells were transformed with pD434-SR-WSP and grown in 1.0 L Terrific Broth media containing 100 μg/mL ampicillin at 37 °C and 200 rpm until an OD_600_ of 0.8. Protein expression was induced by 0.5 mM IPTG at 18 °C for 24 h. The bacterial cell pellet was lysed in phosphate-buffered saline (PBS) containing an additional 150 mM NaCl, 10% glycerol, 2 mM MgCl_2_, 0.5 mg/mL lysozyme, 2.0 μL Benzonase (250 U/μL), 1 mM PMSF, and 2× protease inhibitor tablets (EDTA-free; Roche). Cells were homogenized and bacterial inclusion bodies were harvested by centrifugation. Inclusion bodies were washed 3 times in chilled PBS containing 1% Triton X-100 (*v*/*v*), then resuspended in PBS containing 8 M urea/10 mM imidazole and filtered through a 0.22-μm low protein binding syringe filter (Acrodisc, Pall Corporation, Port Washington, NY, USA). Protein purification was performed by standard denaturing IMAC (Bio-Rad, Hercules, CA, USA), using a 5-mL HisTRAP fast flow column (Sigma Aldrich, St. Louis, MO, USA) connected to a Bio-Rad Profinia chromatography workstation (Bio-Rad, Hercules, CA, USA). The denatured IMAC elution fraction was refolded by dialysis against 500 mL of PBS + 4 M urea (6 h, 4 °C), PBS + 2 M urea (overnight at 4 °C), and then PBS + 1 M urea (6 h, 4 °C). The final purified WSP_33-201_ protein concentration was 2.54 mg/mL.

The tetanus toxoid protein (Pfizer Animal Health, Geelong, Australia) used as a control antigen was a kind gift from Dr. Brendon Chua (University of Melbourne).

### 4.4. Anti-WSP Antibody

Polyclonal anti-WSP was generated and purified by the Walter and Eliza Hall Antibody Facility (WEHI; Melbourne, Australia). Briefly, 2 rabbits (R1881 and R1882) were immunized with 200 μg *w*Mel WSP_33-201_ + complete Freund’s adjuvant, followed by 5 × monthly immunisations with 200 μg *w*Mel WSP_33-201_ + incomplete Freund’s adjuvant. Serum was collected and tested by ELISA pre-immunization, and after the 3rd, 4th, 5th, and 6th immunisations. Polyclonal antibodies were affinity purified using Protein A sepharose. Anti-WSP polyclonal antibody from the final bleed of R1882 was shown to have a strong reactivity to *w*Mel WSP_33-201_ and was therefore selected for use as a positive serological signal control in our experimental ELISAs. All animal experiments were performed in accordance with ethics criteria as set out by the WEHI Animal Ethics Committee.

### 4.5. Wolbachia Detection by Western Blot and qPCR

Aag2 cells with *w*Mel or *w*MelPop, or without *Wolbachia* [60,61], were lysed directly in reducing Laemmeli buffer (Bio-Rad, Hercules, CA, USA) and run on a 12% SDS-PAGE. Proteins were transferred to PVDF membrane, blocked with 5% skim milk in PBS, and then probed with rabbit polyclonal anti-WSP (R1882, 1:1000) and mouse anti-alpha tubulin (Sigma Aldrich, St. Louis, MO, USA; 1:5000). HRP-conjugated anti-rabbit and anti-mouse secondary antibodies (Promega, Madison, WI, USA; 1:2000) were applied, and the blots imaged by enhanced chemiluminescence.

Concurrently, triplicate cell samples were taken from the same flask and analysed by qPCR to quantify the *Wolbachia* density (number of *Wolbachia* per Aag2 cell) using the single copy gene *WSP*. This was done as previously described [62], except qPCR was performed with 3 μL of supernatant, using LightCycler 480 Probes Master kit (Roche, Basel, Switzerland). Probe and primer sequences: *WSP* detection—*WSP* F (5′-CATTGGTGTTGGTGTTGGTG) *WSP* R (5′-ACACCAGCTTTTACTTGACCAG), and *WSP* LC640 probe (5′-TCCTTTGGAACCCGCTGTGAATGA-IowaBlackRQ). Housekeeping gene, *RPS17*—*RPS17* F (5′-TCCGTGGTATCTCCATCAAGCT), *RPS17* R (5′-CACTTCCGGCACGTAGTTGTC), and *RPS17* FAM probe (5′-CAGGAGGAGGAACGTGAGCGCAG-BHQ1) [63]. Relative quantification of *Wolbachia* number per cell was determined using the delta CT method (2^CT(reference)^/2^CT(target)^).

### 4.6. Wolbachia Detection by Immunofluoresence

Aag2 cells with *w*Mel or *w*MelPop, or without *Wolbachia*, were seeded on poly-L-Lysine coated coverslips (ProSciTech, Queensland, Australia) in a 12-well plate. Cells were left to settle for 2 days. Media was removed, and the cells were washed 1 × with PBS, then fixed in 4% PFA/PBS for 10 min. Cells were washed 3 × with PBS, then permeabilized with 0.1% triton-x 100 for 5 min. Cells were washed 3 × with PBS, then blocked with 1% BSA/PBS for 1 h. Coverslips were incubated with rabbit anti-WSP (1:1000) in 1% BSA/PBS for 1 h in a humidified chamber. Cells were washed 3 × with PBS, then incubated with Alexafluor anti-rabbit 594 (1:1000) in 1% BSA/PBS for 1 h in a humidified chamber. Cells were washed 3 × with PBS, then 1 × with milliQ water. Coverslips were mounted in a drop of ProLong Gold antifade mountant with DAPI. Images were captured on a fluorescence Zeiss Imager A1 microscope (ZEISS, Göttingen, Germany) using a 40 × objective and prepared using Fiji software (version 1.52i, Fiji, Madison, WI, USA).

### 4.7. Enzyme-Linked Immunosorbent Assay (ELISA)

All plasma samples were tested for immunoreactivity towards WSP as well as a control antigen, tetanus toxoid protein. Vietnam has a strong vaccination program that includes vaccinating against tetanus from 1 year [21]. We reasoned that all study participants had been previously immunized with this antigen at some time, and that the detection of antibodies against tetanus toxoid would demonstrate the integrity of each plasma sample.

Flat-bottom 96-well Nunc Maxisorb plates (Thermo Fisher Scientific, Waltham, MA, USA) were coated with either recombinant WSP or tetanus toxoid protein (5 μg/mL) in PBS overnight at 4 °C. Plates were washed three times with PBS containing 0.05% Tween-20 (PBST), then blocked with 10% BSA in PBS for 1 h at room temperature. Blocking solution was removed, and plates were washed twice with PBST and once with PBS. Plasmas were diluted 1:100 in 5% BSA in PBST, then serially diluted 7 times at 0.5log_10_ dilutions (in 5% BSA in PBST).

Each WSP-coated plate included paired plasmas from 5 individual participants (taken pre- and post-release of *Wolbachia*-*Ae. aegypti*), a negative control plasma pool (a pool of 5 × Australian Red Cross donors from southern regions of Australia where *Ae. aegypti* are not found), and a positive serological signal control (negative control plasma pool spiked with polyclonal anti-WSP, 1:1000). Tetanus-toxoid-coated plates were similarly assayed with the same paired plasma samples, a negative control (PBS), and a positive signal control (the same pool of 5 × Australian Red Cross donors from southern regions of Australia used above).

In addition to the 105 TNI participants, plasma samples from 29 Red Cross blood donors located in the Australian states of Victoria or South Australia were included as individual negative controls for *Wolbachia* immunoreactivity. These were also assayed in parallel for reactivity to tetanus toxoid.

All plasmas were incubated at room temperature for 2 h and then washed (×6) with PBST. Bound antibodies were detected using HRP-conjugated goat anti-human IgA/G/M (Thermo Fisher Scientific, Waltham, MA, USA) or goat anti-rabbit IgG (for the positive anti-WSP control wells; Promega, Madison, WI, USA). Secondary antibodies were diluted 1:1000 in 5% BSA in PBST and incubated for 1 h at room temperature. Plates were washed (×6) with PBST, then incubated with 3,3′,5,5′-Tetramethylbenzidine (TMB) for ~2 min. The reaction was stopped by addition of 0.1 M HCl. The absorbance of each well was measured at 450 nm (signal) and 620 nm (background) using a BioTek Gen5 microplate reader (Agilent, Santa Clara, CA, USA).

The background-subtracted absorbance values were normalised to the maximum signal of the positive control on that plate (i.e., the pool of 5 × Australian Red Cross plasma samples spiked with rabbit anti-WSP polyclonal antibody for the WSP-coated plates, and unspiked pooled Australian Red Cross plasmas for the tetanus toxoid-coated plates). These normalised values were then graphed against the reciprocal of the dilution for each plasma sample. The antibody titre for each sample was expressed as the reciprocal of the dilution of plasma required to achieve an absorbance value of 0.4 (determined by nonlinear regression analysis performed using GraphPad Prism^®^ software PRISM 7 version, GraphPad Software, Inc., San Diego, CA, USA) [64]. All plasma samples achieved a normalised absorbance value of >0.4 when assayed against tetanus toxoid, and therefore all samples were included in the analyses.

## Data Availability

The raw data generated in this study are available on request from the corresponding author.

## Supplementary Materials

The following supporting information can be downloaded at: https://www.mdpi.com/article/10.3390/pathogens11050535/s1, Figure S1: Expression of recombinant WSP, and anti-WSP reactivity to *w*Mel and *w*MelPop-CLA WSP. Figure S2: Anti-WSP plasma titres from individuals pre- and post-release of *w*MelPop-CLA-*Ae. aegypti*.
