# Supplementary material for: Transient Introgression of Wolbachia into Aedes aegypti Populations Does Not Elicit an Antibody Response to Wolbachia Surface Protein in Community Members
Source: Pathogens. 2022 May 3;11(5):535. doi: 10.3390/pathogens11050535 (PMC9142965; doi:10.3390/pathogens11050535)
Supplement: Supplementary file 1 [file pathogens-11-00535-s001.zip › pathogens-1684445-supplementary materials-conversion update.pdf]

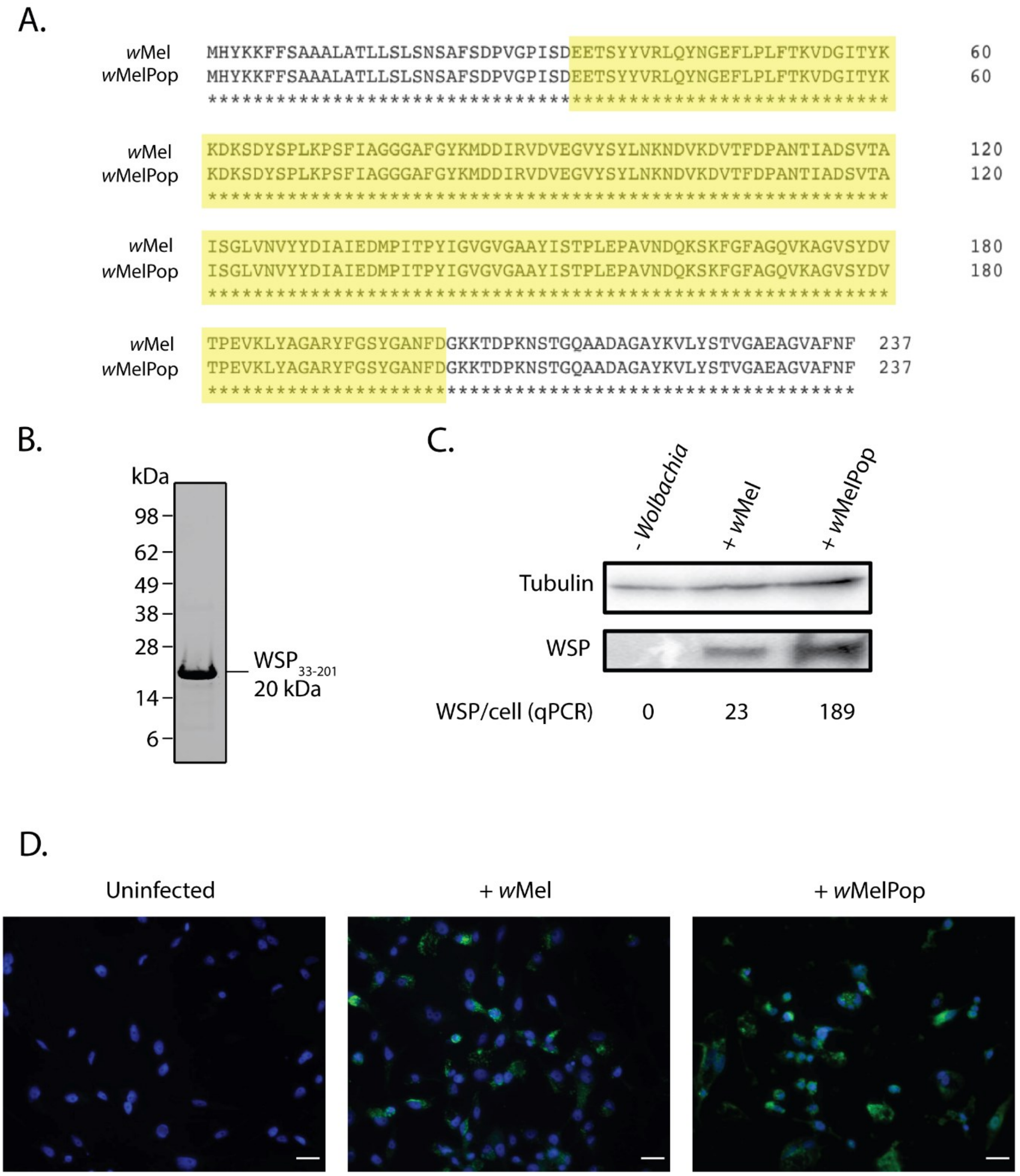

Figure S1. Expression of recombinant WSP, and anti-WSP reactivity to *w*Mel and *w*MelPop-CLA WSP. (**A**) WSP protein alignment from *w*Mel (GenBank ID AE017196.1, protein ID AAS14719.1) and *w*MelPop (GenBank ID NZ_KI440885.1, protein ID WP_010963018.1). The highlighted region indicates the expressed recombinant antigen sequence. (**B**) WSP residues 33-201 ($WSP_{33\text{-}201}$) were expressed and purified as a His-tagged monomeric protein from *E. coli*. Purified protein (12 µg) was run on non-reducing SDS-PAGE and stained with

Coomassie blue. (**C**) Polyclonal anti-WSP raised in rabbits to recombinant WSP_{33-201} demonstrates reactivity to WSP from both *w*Mel and *w*MelPop *Wolbachia* strains (protein lysates from *Wolbachia*-Aag2 cell lines) but not to *Wolbachia*-uninfected Aag2 cell controls. WSP DNA copies per cell were determined by qPCR from the same passage (indicated below Western blot). (**D**) *Ae. aegypti*-derived Aag2 cells, with no *Wolbachia*, *w*Mel, or *w*MelPop were fixed and stained with polyclonal anti-WSP. Coverslips were mounted in ProLong Gold Antifade mountant with DAPI to visualise nuclear DNA and viewed using a Zeiss Imager.A1 microscope. Images and scale bars (20 µm) were generated in Fiji software.

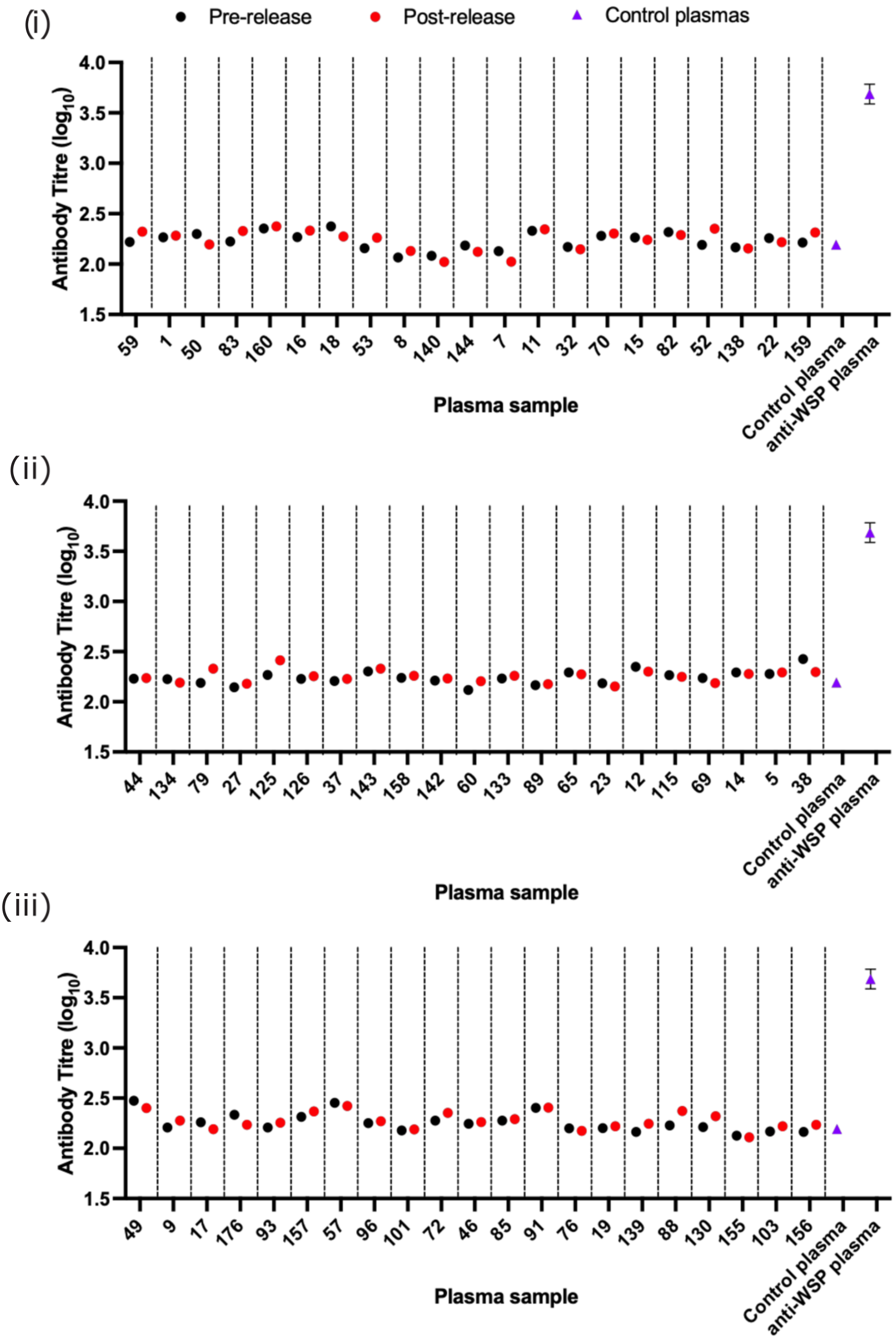
(i)
Pre-release
Post-release
Control plasmas
Antibody Titre (log10)
4.0
3.5
3.0
2.5
2.0
1.5
59 1 50 83 160 16 18 53 8 140 144 7 11 32 70 15 82 52 138 22 159
Control plasma
anti-WSP plasma
Plasma sample
(ii)
Antibody Titre (log10)
4.0
3.5
3.0
2.5
2.0
1.5
44 134 79 27 125 126 37 143 158 142 60 133 89 65 23 12 115 69 14 5 38
Control plasma
anti-WSP plasma
Plasma sample
(iii)
Antibody Titre (log10)
4.0
3.5
3.0
2.5
2.0
1.5
49 9 17 176 93 157 57 96 101 72 46 85 91 76 19 139 88 130 155 103 156
Control plasma
anti-WSP plasma
Plasma sample

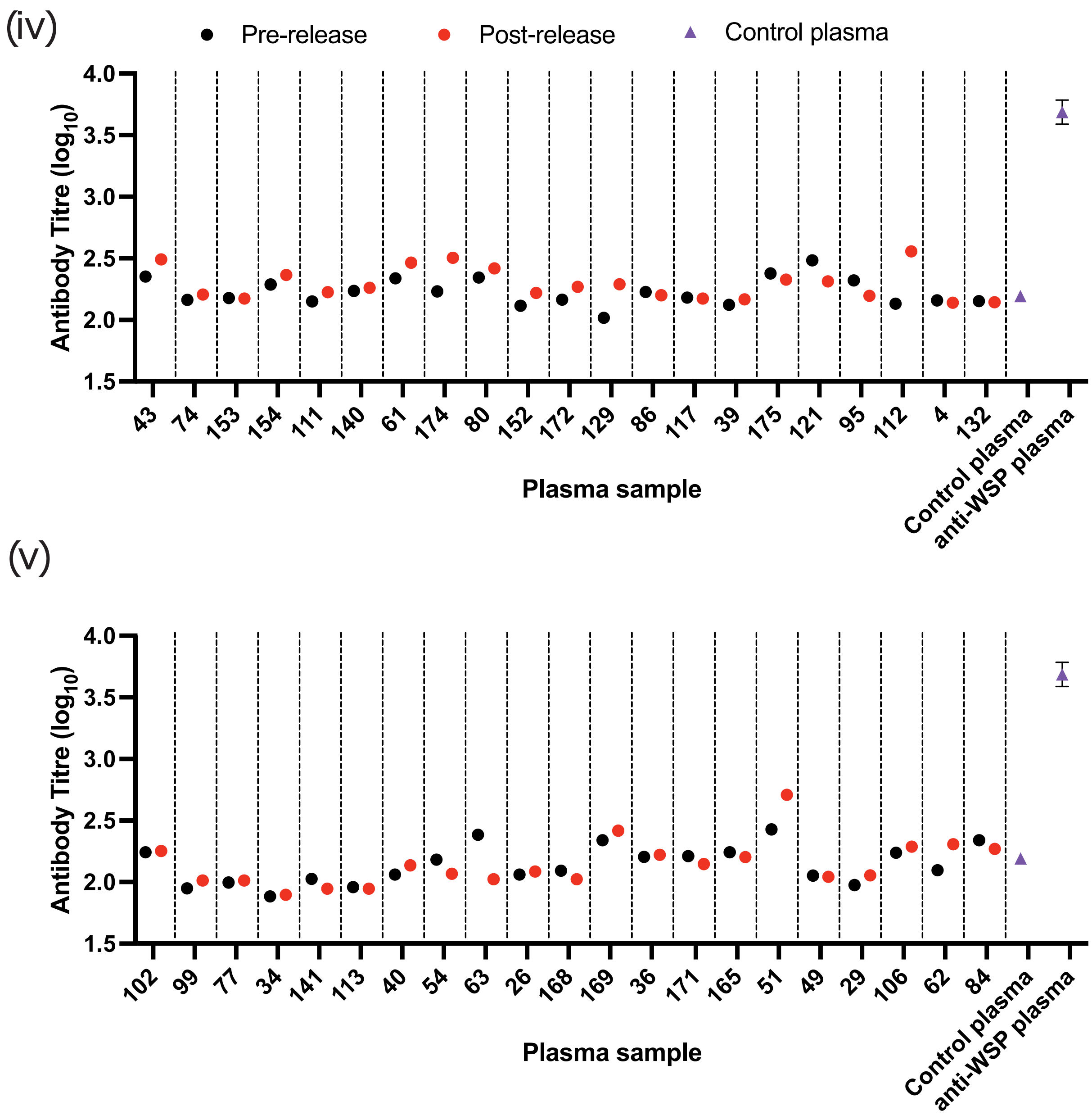

Figure S2. Anti-WSP plasma titres from individuals pre- and post-release of *w*MelPop-CLA-*Ae. aegypti*. (**i**–**v**) Data are a breakdown of anti-WSP plasma titres as shown in Fig. 3, where individual titres from each of the 105 donors living on TNI, Vietnam, pre- and post-release of *w*MelPop-*Ae. aegypti* are shown. Control plasma is a pool of 5 negative control plasmas (from Australian Red Cross donors located in southern regions of Australia), whilst anti-WSP plasma is the negative control plasma pool spiked with polyclonal anti-WSP.
